# Mitochondrial Protein Cox7b Is a Metabolic Sensor Driving Brain-Specific Metastasis of Human Breast Cancer Cells

**DOI:** 10.3390/cancers14184371

**Published:** 2022-09-08

**Authors:** Marine C. N. M. Blackman, Tania Capeloa, Justin D. Rondeau, Luca X. Zampieri, Zohra Benyahia, Justine A. Van de Velde, Maude Fransolet, Evangelos P. Daskalopoulos, Carine Michiels, Christophe Beauloye, Pierre Sonveaux

**Affiliations:** 1 Pole of Pharmacology and Therapeutics, Institut de Recherche Expérimentale et Clinique (IREC), Walloon Excellence in Life Sciences and Biotechnology (WELBIO), Université Catholique de Louvain (UCLouvain), Avenue Hippocrate 57 Box B1.53.09, 1200 Brussels, Belgium; 2 URBC-NARILIS, University of Namur, 5000 Namur, Belgium; 3 Pole of Cardiology, Institut de Recherche Expérimentale et Clinique (IREC), Université Catholique de Louvain (UCLouvain), Avenue Hippocrate 55 Box B1.55.05, 1200 Brussels, Belgium; 4 Walloon Excellence in Life Sciences and Biotechnology (WELBIO) Research Institute, 1300 Wavre, Belgium

**Keywords:** breast cancer, brain metastasis, tissue-specific metastasis, organotropism, cancer metabolism, oxidative phosphorylation (OXPHOS), mitochondria, cyclooxygenase 7b (Cox7b)

## Abstract

**Simple Summary:**

What controls organotropism during cancer metastasis is still largely unknown. The “seed-and-soil hypothesis” of Stephen Paget (1889) proposes that metastatic onset strictly depends on a match between the needs of a given metastatic progenitor cell (the seed) and the resources provided by a given organ (the soil). Here, we decided to challenge this old theory in the context of cancer metabolism. Considering that metastasis can be prevented, we focused on triple-negative breast cancer brain metastasis. Comparing RNAseq data from wild-type human cancer cells and two independent brain-seeking variants, we identified cyclooxygenase 7b (Cox7b) in Complex IV of the mitochondrial electron transport chain as a driver of triple-negative breast cancer brain metastasis. Cox7b is not an easy therapeutic target and is most probably not unique in driving brain metastasis. Therefore, our general approach could be used to identify other metabolic proteins responsible for organotropism and amenable for metastasis-prevention therapy.

**Abstract:**

Distant metastases are detrimental for cancer patients, but the increasingly early detection of tumors offers a chance for metastasis prevention. Importantly, cancers do not metastasize randomly: depending on the type of cancer, metastatic progenitor cells have a predilection for well-defined organs. This has been theorized by Stephen Paget, who proposed the “seed-and-soil hypothesis”, according to which metastatic colonization occurs only when the needs of a given metastatic progenitor cell (the seed) match with the resources provided by a given organ (the soil). Here, we propose to explore the seed-and-soil hypothesis in the context of cancer metabolism, thus hypothesizing that metastatic progenitor cells must be capable of detecting the availability of metabolic resources in order to home in a secondary organ. If true, it would imply the existence of metabolic sensors. Using human triple-negative MDA-MB-231 breast cancer cells and two independent brain-seeking variants as models, we report that cyclooxygenase 7b (Cox7b), a structural component of Complex IV of the mitochondrial electron transport chain, belongs to a probably larger family of proteins responsible for breast cancer brain tropism in mice. For metastasis prevention therapy, this proof-of-principle study opens a quest for the identification of therapeutically targetable metabolic sensors that drive cancer organotropism.

## 1. Introduction

Entry in the metastatic phase often represents a point of no return for cancer patients, as it is associated with a transition from curative to palliative care. This is mainly due to a limitation of therapeutic options, as local treatments are not always an option for (poly)metastatic patients, and secondary tumors are generally more resistant to treatments than primary tumors [1]. Treating brain metastasis offers the additional challenge of blood–brain barrier (BBB) protection in an initially immunopreserved environment [2]. Consequently, poorly symptomatic cancers that are often detected at an advanced, post-metastatic stage are associated with poor 5-year overall survival rates, while slowly evolutive cancers that are commonly detected at the premetastatic stage are, in contrast, associated with much longer patient survival rates. Between these two extremes, improvements of detection methodologies and their systematic use in individuals at risk allow an early detection of some aggressive types of cancers. This is typically the case of triple-negative breast cancer (TNBC), which is most often detected at the premetastatic stage [3]. However, these cancers usually evolve quite rapidly despite early treatment delivery, and distant metastases have a high prediction to occur. About 35% of TNBC patients develop metastases in the course of the disease, despite surgery, chemotherapy, and radiotherapy [4]. Furthermore, immunotherapy with checkpoint inhibitors has only shown modest efficacy in a subset of patients [5]. Overall, polymetastatic TNBC is currently still regarded as a largely incurable disease [6].

While the vast majority of solid tumors primarily colonize regional lymph nodes, distant metastases do not occur randomly: different cancer types present different metastatic patterns, with different frequencies. For example, malignant melanomas preferentially metastasize to the lungs (frequency of 85%, including micrometastases), the liver (54–77%), the gastrointestinal tract (~60%), the brain (36–54%), the bones (23–49%), and distant skin and subcutis (~18%) [7,8,9]. In the case of TNBC, metastases occur in the central nervous system (~46% of patients), lungs (~41%), liver (~29%), bones (~24%), and breast or chest wall (~22%) [10]. Brain metastasis is particularly detrimental, with a median patient survival time of 4 to 7 months [10,11]. These numbers further highlight that several organs can be colonized at the same time. Thus, in both cases, a large proportion of patients that were metastasis-free at diagnosis become polymetastatic over the course of the diseases.

Among circulating tumor cells (CTCs), only a minority, termed “metastatic progenitor cells” [12,13], possess all the characteristics that are mandatory to successfully establish a metastasis. The preference of these cells for a limited panel of secondary organs has been theorized by Stephen Paget [14] in 1889 and revisited several times since then (see reference [15] for a recent review). In the “seed and soil” hypothesis, Paget proposed that metastases do not occur randomly in secondary organs but, rather, colonize a given organ based on a match between the needs of a (sub)population of metastatic progenitor cells (“the seed”) and the resources provided by the secondary organ (“the soil”). His proposition highlighted the importance of the tumor microenvironment at both primary and secondary tumor locations. Further interpretation also suggests the possible coexistence of several different populations of metastatic progenitor cells with different tropisms for secondary organs in the same patient.

So, what does the seed-and-soil hypothesis implicate? On the one hand, the capabilities of metastatic progenitor cells would depend on the genetic and epigenetic characteristics inherited from their tissue of origin as well as from genetic and epigenetic changes acquired over time, from the onset of malignancy until dissemination. Indeed, metastatic progenitor cells are, nowadays, consensually believed to originate from metabolically hostile cancer areas characterized by a combination of hypoxia, nutrient deprivation, and metabolic waste accumulation causing, e.g., microenvironmental acidification [16,17,18,19,20,21,22,23]. Each parameter, per se, contributes to the metastatic switch, defined as a discrete event converting nonmetastatic cancer cells to their metastatic version [24]. However, it is the coexistence of these parameters and their fluctuation over time and space that constitutes an ideal situation for cancer cell adaptation and evolution, which both necessitate environmental changes to operate [25]. The outcome of these processes is the generation of subsets of cancer cells that simultaneously possess mesenchymal characteristics, resist anoïkis, migrate and invade directionally, resist redox and shear stresses in the systemic circulation, and possess stem cell characteristics [26]. On the other hand, resources available at the secondary site would depend not only on the basal composition and mode of function of the organ to be colonized but also on the organ’s “education” by the primary tumor, a paradigm formulated as “premetastatic niche” formation [27]. In the process, cancer cells at the initial site produce and secrete soluble factors (including, e.g., VEGF-A, PLGF, G-CSF, CCL2, TGFβ, TNF, and enzymes such as lysyl oxidase that remodel the extracellular matrix) and exosomes that modify the composition and integrity of the secondary site (see reference [28] for a detailed review). As a result, vascular leakiness is increased; resident cells, such as fibroblasts, modify their behavior; non-resident cells, such as bone-marrow-derived cells and neutrophils, are recruited; a proinflammatory milieu offering immunoprotection is formed; and the structure of the extracellular matrix is remodeled to yield a provisional matrix promoting cell migration, invasion, and, hence, colonization [28].

The homing of metastatic progenitor cells at the secondary site is facilitated by the enhanced permeability of blood vessels lining premetastatic niches. In an active process, CTCs expressing E-selectin ligands (that may include CD44, PSGL-1, ESL-1, β2-integrins, and L-selectin [29]) roll on and adhere on endothelial cells expressing E-selectin at their luminal surface, extravasate (which may require proteolytic activities by CTCs, especially to cross the BBB during brain metastasis [30]), and invade the niche. There, they anchor to host cells and undergo a process of differentiation to generate a secondary tumor. Intravascular metastasis is a rare event.

In the general context of metastasis, we previously observed that mitochondria act as metabolic sensors of the primary tumor microenvironment, producing superoxide and activating the prometastatic transforming growth factor β (TGFβ) pathway in metabolically hostile tumor microenvironments [31]. Inactivating mitochondrial superoxide with specific drugs MitoTEMPO or MitoQ blocked the metastatic process as a whole [13,31,32]. Others showed that transferring superoxide-producing mitochondria from metastatic to nonmetastatic cancer cells was sufficient to turn recipient cells into metastatic progenitor cells [33]. In the context of the seed-and-soil hypothesis, we, therefore, hypothesized that a successful metastatic process would require a match between the metabolic needs of a given subset of metastatic progenitor cells and the availability of specific metabolites at a given secondary site. In other words, we propose that the metabolic preferences of a metastatic progenitor cell would participate in, and perhaps drive, organotropism. Thus, metabolic pairing between different “seeds” and different “soils” would be responsible for the existence of a restricted panel of secondary sites for a given tumor type.

This study on brain tropism is the first of a series addressing this general hypothesis. Using the human TNBC MDA-MB-231 cell line as a working model, we report a cause–effect relationship between the expression of cyclooxygenase 7b (Cox7b) in Complex IV of the electron transport chain (ETC) of metastatic progenitor cells and the brain tropism of these cells. In brain-seeking variants, Cox7b expression enhances oxidative phosphorylation (OXPHOS), while, compared to other organs, the brain is well-known to be enriched in OXPHOS substrates glucose, lipids, and lactate. Cox7b most probably belongs to a larger family of proteins driving brain metastasis, and other organs would offer other substrates to other subsets of metastatic progenitor cells, which could lead to the potential identification of several targets for the therapeutic prevention of tissue-specific metastasis.

## 2. Materials and Methods

### 2.1. Chemicals and Reagents

Unless stated otherwise, all chemicals and reagents were from Sigma-Aldrich (Overijse, Belgium).

### 2.2. Cells and Cell Culture

Parental MDA-MB-231 human triple-negative breast adenocarcinoma cancer cells were from Caliper (Mechelen, Belgium; catalogue #119369). MDA-MB-231-derived brain-seeking variants 231-BR [34] and 231-BR-2 [35,36] were kind gifts from Patricia Steeg (National Cancer Institute, Bethesda, MD, USA) and Harikrishna Nakshatri (Indiana University School of Medicine, Indianapolis, IN, USA), respectively. Cells were routinely cultured in DMEM containing glutaMAX and 4.5 g/L glucose (Thermo Fisher, Erembodegem, Belgium; catalogue #61965026) supplemented with 10% FBS and maintained at 37 °C in a 5% CO_2_ humidified atmosphere. Cells were authenticated using short tandem repeat (STR) profiling (Eurofins Genomics, Ebersberg, Germany).

Human astrocytes (T0281, expressing hTERT), mouse astrocytes (T0289, expressing the SV40 large T antigen), human hepatocytes (T0063, expressing HPV E6/E7, hTERT and MycT58A), and human bronchial epithelial cells (T0753, expressing hTERT and Cdk4) used in migration assays were immortalized cells from Applied Biological Materials Inc. (ABM, Richmond, BC, Canada). Astrocytes were routinely cultured in DMEM with glutaMAX and 4.5 g/L glucose (Thermo Fisher; catalogue #61965026) supplemented with 10% FBS and 5% astrocyte growth supplement (Sanbio, Uden, The Netherlands; catalogue #1852; for human astrocytes only). Hepatocytes were maintained in PriGrow IX medium (ABM; catalogue #TM019) supplemented with 10% FBS and then were progressively transferred to the same medium as cancer cells. Bronchial cells were maintained in Prigrow X medium (ABM; catalogue #TM0753), and then were progressively transferred to bronchial epithelial cell growth medium (BEGM; Lonza, Verviers, Belgium; catalogue #CC-3170).

### 2.3. Genetic Manipulations

For constitutive luciferase and GFP expression, cells were infected with lentiviruses carrying the luciferase and GFP sequences along with puromycin resistance gene (Amsbio, Alkmaar, The Netherlands; catalogue #LPV020), as detailed in Appendix A.1.

*COX7B* gene silencing was performed using a CRISPR-Cas9 strategy following Zhang’s lab protocol [37], and Cox7b overexpression using the pCMV3 expression vector (Bio-Connect, Te Huissen, The Netherlands; catalogue #HG20762-UT), as detailed in Appendix A.2. and Appendix A.3. pSpCas9(BB)-2A-Puro (PX459) V2.0 and pU6-(BbsI) CBh-Cas9-T2A-mCherry were kind gifts from Feng Zhang (Addgene, Watertown, MA, USA; plasmid #62988) and Ralf Kuehn (Addgene; plasmid #64324), respectively.

### 2.4. Metastatic Take in Mice

On day 0, 6-week-old female NMRI nude mice (Janvier, Le Genest-Saint-Isle, France) received image-guided intraventricular injections of 100,000 cancer cells, using a Vevo 2100 imaging system (FUJIFILM VisualSonics, Toronto, ON, Canada) equipped with a 30 MHz transducer. Briefly, mice were anesthetized (80 mg/Kg ketamine and 8 mg/Kg xylazine) and secured on the animal platform in supine position, and their thoraxes were shaved. Two-dimensional (2D) parasternal long-axis ultrasound images of the left ventricle were acquired, in order to ascertain the optimal point in the apex of the heart for the intraventricular injection. A microinjector system with a 26G hypodermic needle was used to perform a precise echocardiography-guided intraventricular injection of 100,000 cancer cells constitutively expressing luciferase and GFP. Following injection, the blood flow within the left ventricle was closely observed (and images were recorded), confirming that the cancer cells were successfully injected intraventricularly. All mice were followed-up for a few minutes with echography, to ascertain that the injection did not lead to any injury, and were closely monitored until recovery.

Metastasis development was monitored using a Xenogen IVIS 50 bioluminescence imaging system (PerkinElmer, Seer Green, UK) and quantified with Living Image software (PerkinElmer). Every week, mice were injected i.p with 0.15 mg/g bodyweight of luciferin (PerkinElmer) and were anesthetized using isoflurane after a 10-min incubation. Chemiluminescence was detected with a 1–12 s acquisition time. Mice were sacrificed after 4 weeks by cervical dislocation under terminal anesthesia, and organ chemiluminescence was acquired ex vivo before fixation in 4% paraformaldehyde (PFA).

### 2.5. Cell Migration and Invasion

Corning transwell inserts (Avantor, Leuven, Belgium; catalogue #62406-198) were used to measure cell migration and invasion capacities, as detailed in Appendix A.4. FBS 1% (general migration/invasion) or confluent nonmalignant cells (astrocytes, hepatocytes, bronchial cells) seeded in the lower chamber were used as chemoattractants. 

### 2.6. Cell Numbers

Cell numbers were determined over time on a SpectraMax i3 spectrophotometer equipped with a MiniMax imaging cytometer (Molecular Devices, Munich, Germany), after seeding 5000 cells per well in a 96-well plate. Results were normalized to initial cell numbers.

### 2.7. Metabolic Assays

Oxygen consumption rates (OCRs) were measured on a Seahorse XF96 bioenergetics analyzer using the XF Cell Mito Stress Test Kit and the Fuel Flex test kit, in accordance with the instructions of the manufacturer (Agilent, Machelen, Belgium). Details are provided in Appendix A.5. Glucose and lactate concentrations were measured using an enzymatic CMA600 analyzer (Aurora Borealis, Schoonebeek, The Netherlands), in accordance with the instructions of the manufacturer, in the supernatant of 150,000 (for 24 h assays) and 250,000 (for 48 h assays) cells seeded in exactly 1 mL of culture medium. Wells containing medium only were used as controls for the calculation of glucose consumption and lactate production. The ATP content of 10,000 cells per well (96-well plate) was measured using the Cell titer Glo assay of Promega (Leiden, The Netherlands; catalogue #G7570). All metabolic measurements were normalized to total protein content determined after overnight incubation with 0.5 M NaOH using the Bio-Rad protein assay (Temse, Belgium; catalogue #5000006) on a SpectraMax i3 spectrophotometer equipped with a MiniMax imaging cytometer.

### 2.8. Electron Microscopy

Cells were collected and resuspended in 400 μL of a 2.5% glutaraldehyde solution containing 0.1 M of sodium cacodylate at pH 7.4 in a pyramidal BEEM capsule (Agar Scientific, Stansted, UK; catalogue #G360). Samples were then processed as previously described [38]. Images were acquired on a TECNAI G² 20 LaB6 transmission microscope (Field Electron and Ion Company, Hillsboro, OR, USA).

### 2.9. Mitochondrial Abundance and Mitochondrial DNA Content

Mitochondrial DNA (mtDNA) content was measured using RT-qPCR as previously described [39]. Briefly, total DNA was isolated with a QIAmp DNA kit (Qiagen, Antwerp, Belgium). The 12S-rRNAA mitochondrial gene (forward primer: 5′-GTA CCC ACG TAA AGA CGT TAG G-3′; reverse primer: 3′-TAC TGC TAA ATC CAC CTT CG-5′; labeled probe: 5′-CCC ATG AGG TGG CAA GAA AT-3′ FAM), in parallel with nuclear gene RNAseP (RNAseP VIC-labeled probe; Thermo Fisher Scientific; catalogue #4401631), were then analyzed by RT-qPCR (50 ng of sample and 1 µL of each primer pair [10µM]), with TaqMan universal master mix II with UNG (Thermo Fisher). For presentation, mtDNA content was normalized to nuclear DNA (nDNA) content [40].

### 2.10. Microarray Database Analysis

According to Gene Expression Omnibus (GEO) and reference [36], database #GSE66495 reports on the whole genome expression, determined using Illumina Human HT-12 V4 expression beadchips, of MDA-MB-231 parental cells and tissue-specific metastatic variants derived thereof (including 231-BR), which were maintained in MEM with 10% FBS. For database reanalysis, we first extracted metabolic genes related to glycolysis, OXPHOS, and the TCA cycle. We next retained only genes that were differentially expressed (*p* < 0.05, using one-way ANOVA) in brain (231-BR), adrenal (ADMD-231), bone (BMD-231) and/or lung (LMD-231) metastatic variants, compared to parental MDA-MB-231 cells. They are displayed in Table 1. We then identified the genes that were differently expressed (*p* < 0.05, using one-way ANOVA) in 231-BR versus ADMD-231, BMD-231, and LMD-231 cells. Expression changes were independently confirmed by RT-qPCR using fresh lysates from brain-seeking variants 231-BR and 231-BR-2 and MDA-MB-231 parental cells.

### 2.11. Real-Time Quantitative PCR

Total RNA was collected using the NucleoSpin RNA kit (Filter Service, Eupen, Belgium; catalogue #740955.50), quantified with a NanoDrop 1000 Spectrophotometer (Thermo Fisher), and reverse-transcribed in cDNA with the RevertAid First Strand cDNA synthesis kit (Thermo Fisher; catalogue #K1621), using the same quantity (500–1000 ng) for all RNA samples and a 90-min incubation time. cDNAs were diluted 1:10 in DNase/RNase-free distilled water (Thermo Fisher), and 2 µL were used with 5 µL of 2X Takyon qPCR Master Mix and 0.2 µL of each primer (10 µM) and completed to 10 µL with water for RT-qPCR analysis (ViiA 7417 Real-Time instrument, Thermo Fisher). Primers were: *ALDH9A1* Forward 5′-AAG GAG CAG GGT GCT AAA GT-3′ and Reverse 5′-TCG TCT CTG CAA TTA GTT AAT ACA C-3′; *FH* Forward 5′-TGC CAA CCC CAG TTA TTA AAG C-3′ and Reverse 5′-CTT CAG CTA CCT CAT CTG CTG-3′; *NDUFB8* Forward 5′-CGG ATG ATG GCA TGG GGT A-3′ and Reverse 5′-GGT GCC AGT GCA TCG GTT-3′; *COX7B* Forward 5′-TAC CTG AAG CGA ATT GGC AC-3′ and Reverse 5′-GCT TCG AAC TTG GAG ACG AT-3′; and *β-actin* Forward 5′-CCC GCG AGC ACA GAG C-3′ and Reverse 5′-TCA TCA TCC ATG GTG AGC TGG- 3′. All gene expression data were normalized to *β-actin* gene expression. 

### 2.12. Western Blotting

Western blotting was performed, as previously described [41], after protein collection in RIPA buffer containing phosphatase (PhosSTOP) and protease (proteases inhibitor cocktail) inhibitors. Membranes were incubated overnight with primary rabbit antibodies against ALDH9A1 (Proteintech; catalogue #26621), FH (BIOKE, Leiden, The Netherlands; catalogue #4567S), NDUFB8 (Proteintech, Manchester, UK; catalogue #14794), COX7b (Abcam, Cambridge, UK; catalogue #ab137094), and Vinculin (BIOKE; catalogue #4650S), or mouse antibodies against β-actin (Sigma-Aldrich; catalogue #A5441). Staining was revealed with an Amersham Imager 600 (Diegem, Belgium). All data are normalized to vinculin or β-actin expression.

### 2.13. Immunohistochemistry

Brains were collected, cut along the separation between right and left hemispheres, and embedded in paraffin. Sections (5 μm thick) were performed from the center of each hemisphere, to produce 10 slides for each sample. For each hemisphere, 3 slides from the beginning, the middle, and the end were used for immunostaining, and the process was repeated up to 3 times in order to analyze slices representative of the whole brain.

Brain sections were immunostained for GFP (Bio-Techne, Abingdon, UK; catalogue #600-308; BIOKE; catalogue #2956), with a secondary Envision anti-rabbit antibody coupled to HRP (Agilent; catalogue #K4003), and hematoxylin and eosin counterstained. Slides were scanned at 20x magnification with a SCN400 bright field Slide Scanner (Leica Biosystems, Diegem, Belgium). Metastasis number and surface area were determined using cytomine (Liège, Belgium; cytomine.org; accessed on 1 December 2021) and QuPath software version 0.1.2 (Belfast, UK) [42].

### 2.14. Clinical Database Analysis

Overall Survival (OS) curves were generated on Kaplan–Meier plotter (kmplot.com) with the auto select best cutoff for the 202110 Affy ID (*COX7B*) on the RNA-seq mRNA dataset (breast cancer and renal clear cell carcinoma in pan-cancer) and on gene chip mRNA datasets for lung cancers [43]. Sources for the databases include GEO, EGA, and TCGA.

### 2.15. Statistics

Data are shown as means ± SEM (error bars are sometimes smaller than symbols) or as individual values with the median. *n* indicates the total number of replicates per group/condition. Graphpad Prism version 9.2.0. (San Diego, CA, USA) was used for statistical analyses. Mann–Whitney U test, Student’s t-test, one-way ANOVA, and two-ways ANOVA were used where indicated. *p* < 0.05 was considered to be statistically significant.

## 3. Results

### 3.1. Validation of Brain-Seeking Variant Models Derived from Human MDA-MB-231 Triple-Negative Breast Cancer Cells

**Figure 1:**
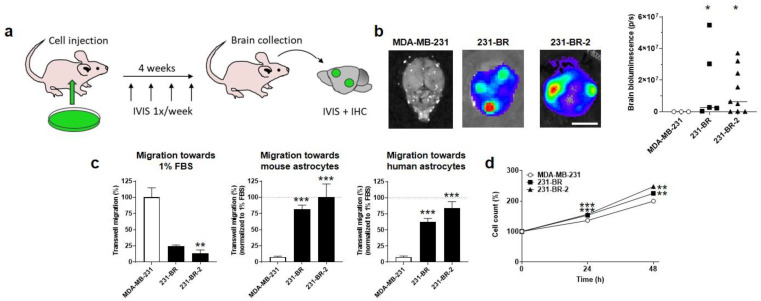
Validation of the brain tropism of MDA-MB-231-derived brain-seeking variants. (**a**) Schematic representation of in vivo experiments, where 6-week-old female mice were injected intracardially with 100,000 luciferase- and green fluorescent protein (GFP)-expressing cancer cells on Day 0, imaged once a week to track metastases, and sacrificed at Week 4 for organ collection, followed by ex vivo bioluminescence imaging and immunohistochemistry. (**b**) Ex vivo bioluminescence imaging of mouse brains at the end of the protocol illustrated in (**a**). Pictures on the left are representative of mouse brains in luciferin-containing medium captured using a Xenogen IVIS 50 bioluminescence imaging system. The right graph shows brain bioluminescence intensity in mice having received parental MDA-MB-231 cancer cells or 231-BR or 231-BR-2 brain-seeking variants (*n* = 3–9). Bar = 5 mm. (**c**) Migration of MDA-MB-231, 231-BR, and 231-BR-2 cells was assayed in transwells towards 1% FBS (*n* = 2–3), towards mouse astrocytes (*n* = 6) or towards human astrocytes (*n* = 6). (**d**) Cell count (%) over time on a SpectraMax i3 spectrophotometer equipped with a MiniMax imaging cytometer, after seeding 5000 cells per well in a 96-well plate (*n* = 18–19). Data are shown as individual values and medians (**b**) or as means ± SEM (**c**,**d**). * *p* < 0.05, ** *p* < 0.01, *** *p* < 0.005; compared to MDA-MB-231; using Mann–Whitney test (**b**), one-way ANOVA with Dunnett’s post hoc test (**c**), or two-way ANOVA with Dunnett’s post hoc test (**d**).

The objective of our study was to identify metabolic protein(s) responsible for the brain tropism of human metastatic breast cancer. As models, we used MDA-MB-231 triple-negative breast cancer (TNBC) cells and two independently derived brain-seeking variant cell lines, 231-BR and 231-BR-2 [34,35,36], which were generated by serial cycles of in vivo selection in mice. The selection protocol involved intracardiac cancer cell injection in the left ventricle of female nude mice, surgical isolation, expansion of metastatic cancer cells retrieved from the brain, and intracardiac injection of these cells sequentially in additional animals for several rounds, until metastatic dissemination became restricted to the brain [34,35,36]. To identify metabolic drivers of brain-specific metastasis, we first ascertained the validity of the two model cell lines in vitro and in vivo. Short tandem repeat (STR) profiling confirmed that all variants were genomically similar to the parental MDA-MB-231 cells (Table S1). For in vivo assays, cells were infected with lentiviruses to constitutively express luciferase and green fluorescent protein (GFP). An intracardiac injection of 100,000 MDA-MB-231 parental cells (Figure 1a) did not generate brain metastases in female nude mice, whereas the use of either the 231-BR or 231-BR-2 variants yielded metastases in most animals (4/5 for 231-BR and 6/9 for 231-BR-2) 4 weeks after injection, which were detected by ex vivo bioluminescence imaging on isolated brains (Figure 1b).

For in vitro model validation, we developed a transwell assay aimed to test the general and organotropic migration of the human breast cancer cells in the upper well towards 1% FBS or towards living immortalized nonmalignant astrocytes in the lower well, respectively. Since the selection of the brain-seeking variants was made in mice, we tested both mouse and human astrocytes as attractants. While parental MDA-MB-231 cells had a higher capacity to migrate towards 1% FBS compared to the two brain-seeking variants, conversely, 231-BR and 231-BR-2 cells migrated much more efficiently towards mouse or human astrocytes (Figure 1c), thus validating their preferential tropism for the brain. Of note, both brain-seeking variants were slightly, yet significantly, more proliferative than the parental cells, as determined by direct cell counting over time (Figure 1d).

### 3.2. Brain-Seeking Variants of MDA-MB-231 Cells Undergo an Oxidative Switch

**Figure 2:**
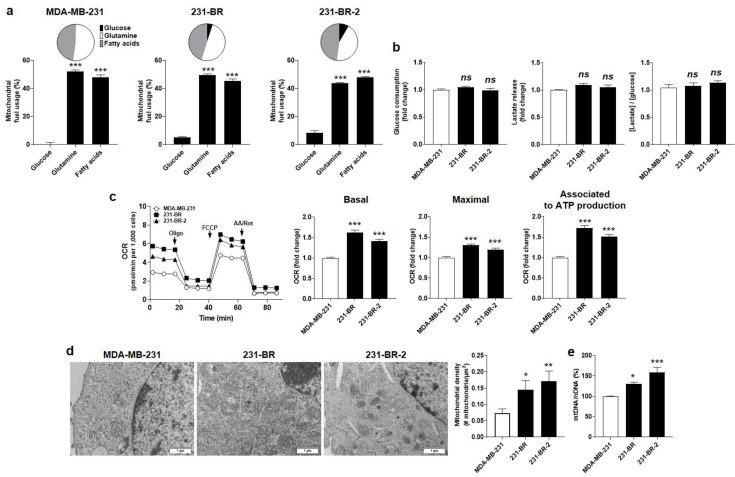
Brain-seeking variants are more oxidative than parental MDA-MB-231 human breast cancer cells. (**a**) Mitochondrial fuel usage of MDA-MB-231 (**left**, *n* = 14), 231-BR (**middle**, *n* = 14–16), and 231-BR-2 (**right**, *n* = 14–15) cancer cells was determined using the Fuel Flex test kit (Agilent) on a Seahorse XF96 bioenergetics analyzer. Data are presented as pie and column graphs, where total fuel usage = 100%. (**b**) Glucose consumption (**left**, *n* = 11–16), lactate production (middle, *n* = 16–17), and the lactate/glucose ratio (**right**, *n* = 13–16) were determined from measurements in deproteinized cell supernatants using an enzymatic CMA600 analyzer. (**c**) Basal (**left**, *n* = 29–32) and maximal (**middle**, *n* = 28–32) oxygen consumption rates (mtOCRs), as well as the OCR of the cells associated to mitochondrial ATP production (**right**, *n* = 29–32), were measured using a XF Cell Mito Stress Test kit (Agilent) on a Seahorse XF96 bioenergetics analyzer. Representative Seahorse traces are shown on far left. (**d**) Transmission electron microscopy pictures of the cells are shown on the left (bars = 1 µm), and the mitochondrial density is quantified on the right graph (*n* = 6–12). (**e**) Mitochondrial DNA/nuclear DNA (mtDNA/nDNA) cell content determined using RT-qPCR (*n* = 5). All data are shown as means ± SEM. * *p* < 0.05, ** *p* < 0.01, *** *p* < 0.005, ns: *p* > 0.05; compared to first column; using one-way ANOVA with Dunnett’s post hoc test (**a**–**c**,**e**).

Using Seahorse oximetry and the dedicated Fuel Flex test kit of Agilent, we next determine the oxidative metabolic preferences of the parental and brain-seeking variant cells. The assay involves sequential inhibition of glucose-fueled (using 2 µM of mitochondrial pyruvate carrier inhibitor UK5099), glutamine-fueled (using 3 µM of glutaminase 1 inhibitor BPTES), and lipid-fueled (using 4 µM of carnitine palmitoyl-transferase 1A inhibitor Etomoxir) OXPHOS. The results show that OXPHOS in MDA-MB-231 cells was supported almost equally by glutamine (52%) and fatty acids (48%), but not at all by glucose, whereas OPXHOS in the two brain-seeking variants was supported not only by glutamine and fatty acids but also by glucose (5.1 ± 0.6 % for 231-BR and 8.4 ± 1.4% for 231-BR-2 cells) (Figure 2a). Glucose uptake, lactate release, and, hence, the glycolytic ratio ([glucose]/[lactate]) were unchanged in full medium (DMEM containing glutaMAX, 4.5g/L glucose, and 10% FBS) (Figure 2b). Therefore, changes in the capacity to use OXPHOS fuels reflected an increased oxidative flexibility of brain-seeking variants compared to parental cells, rather than an increased dependency on glucose. Accordingly, both 231-BR and 231-BR-2 cell lines presented improved basal and maximal respiration activities as well as an improved oxidative ATP production, compared to parental cells (Figure 2c).

The oxidative switch evidenced in brain-seeking variants was linked to qualitative and quantitative changes affecting mitochondria. Qualitatively, electron microscopy revealed enlarged mitochondria in 231-BR compared to the parental cells, whereas they were smaller but more abundant in 231-BR-2 compared to MDA-MB-231 cells (Figure 2d). The mitochondrial to nuclear DNA ratio (mtDNA/nDNA) was determined using RT-qPCR, revealing a significantly increased mtDNA abundance in both 231-BR and 231-BR-2 compared to the parental cells (Figure 2e). Collectively, we concluded that the oxidative efficiency of mitochondria was increased in the brain-seeking variants of MDA-MB-231 cells.

### 3.3. Identification of Four Candidate Metabolic Genes That Could Account for the Brain Tropism of Human Breast Cancer Cells

Based on our working hypothesis of a metabolic preference of brain-seeking variants for metabolites present in the brain and on the above evidence of metabolic differences in the filiation, we next aimed to identify metabolic genes/proteins associated to the brain tropism of 231-BR and 231-BR-2 cells. We first analyzed the publicly available microarray database GEO #GSE66495, reporting on the whole genome expression of not only MDA-MB-231 and 231-BR cells but also MDA-MD-231-derived adrenal (ADMD-231), bone (BMD-231), and lung (LMD-231) metastatic variants [36]. We focused on genes involved in glycolysis, the TCA cycle, and OXPHOS.

Using the two-step methodology described in the materials and methods, we identified 22 metabolic genes differentially expressed in at least one metastatic variant compared to parental MDA-MB-231 cells, among which 6 were further differentially expressed in 231-BR cells compared to any other metastatic variant (Table 1). Significantly upregulated genes were *ALDH9A1* (Genbank ID 223, on chromosome 1) encoding aldehyde dehydrogenase 9 family member A1, *FH* (Genbank ID 2271, on chromosome 1) encoding fumarate hydratase, and *COX7B* (Genbank ID 1349, on chromosome X) encoding cytochrome c oxidase subunit 7B. Significantly downregulated genes were *ALDH1A3* (Genbank ID 220, on chromosome 15) encoding aldehyde dehydrogenase 1 family member A3, *NDUFB8* (Genbank ID 4714, on chromosome 10) encoding NADH:ubiquinone oxidoreductase subunit B8, and *PGM5* (Genbank ID 5239, on chromosome 9) encoding phosphoglucomutase 5.

Among the six genes, *ALDH1A3* and *PGM5* expression was not significantly different (*p* > 0.05) between 231-BR and parental MDA-MB-231 cells (Table 1). They were, therefore, excluded from further analysis.

### 3.4. Cytochrome c Oxidase Subunit 7b in Mitochondrial Complex IV Is a Candidate Protein Supporting the Brain Tropism of Human Breast Cancer Cells

We next aimed to validate our short list of four genes: *ALDH9A1*, *FH*, *NDUFB8*, and *COX7B*. To avoid idiosyncrasies that would have been associated to 231-BR cells, changes in gene expression were independently tested using RT-qPCR in both 231-BR and 231-BR-2 brain-seeking variants. We further verified that the changes in protein matched the changes in mRNA expression. Uncropped western blots are displayed in Figure S1.

**Figure 3:**
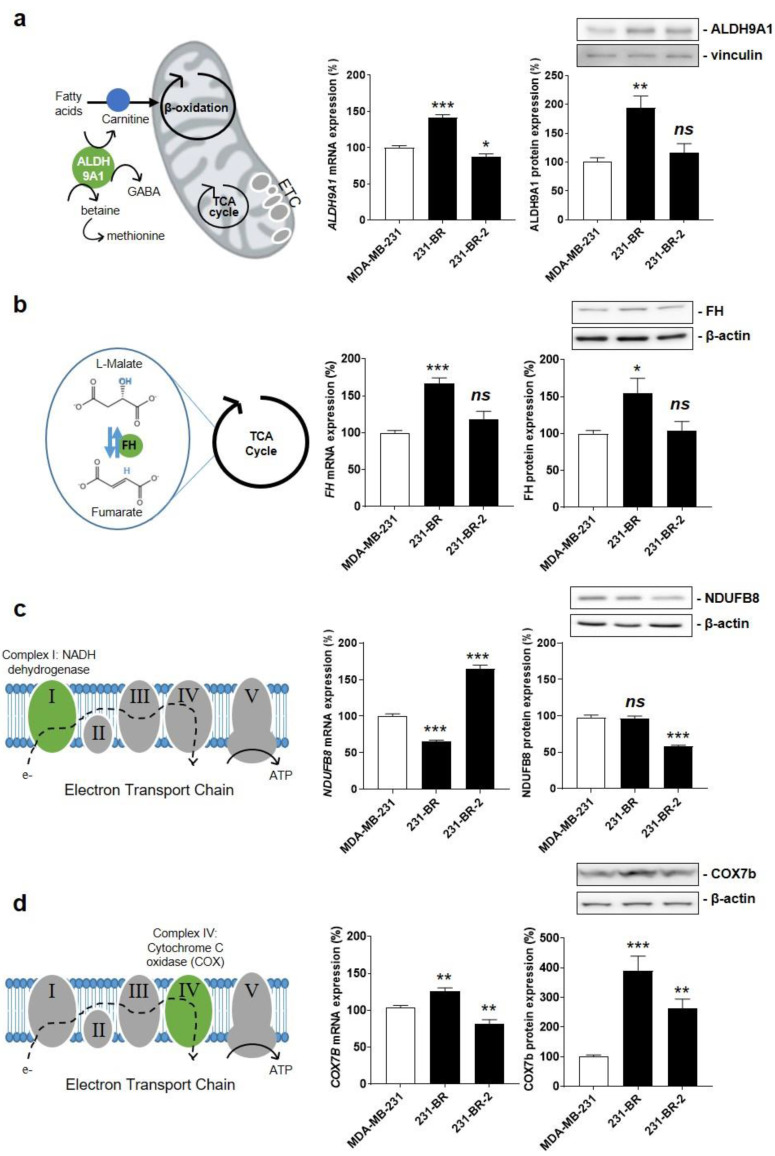
Identification of Cox7b as a candidate protein for the brain tropism of human breast cancer. (**a**) The left drawing depicts the three main functions of aldehyde hydrogenase 9A1 (ALDH9A1) that catalyzes the biosynthesis of γ-aminobutyric acid (GABA) in the cytosol, the biosynthesis of carnitine to facilitate the transport of fatty acids across the inner mitochondrial membrane, and the oxidation of γ-aminobutyraldehyde and aminoaldehydes derived from polyamines in the mitochondrial matrix. The middle graph shows *ALDH9A1* mRNA expression normalized to *β-actin* (*n* = 5–6), and the right graph shows ALDH9A1 protein expression normalized to vinculin (*n* = 6), in MDA-MB-231, 231-BR, and 231-BR-2 cancer cells. (**b**) The left drawing depicts fumarate hydratase (FH) activity, which catalyzes the reversible hydration of fumarate to malate in the TCA cycle. The middle graph shows *FH* mRNA expression normalized to *β-actin* (*n* = 5–6), and the right graph shows FH protein expression normalized to β-actin (*n* = 9). (**c**) The left drawing localizes NDUFB8 as a component of electron transport chain (ETC) Complex I (green). The middle graph shows *NDUFB8* mRNA expression normalized to *β-actin* (*n* = 6), and the right graph shows NDUFB8 protein expression normalized to β-actin (*n* = 3). (**d**) The left drawing depicts cyclooxygenase 7b (COX7b) as a component of ETC Complex IV (green). The middle graph shows *COX7B* mRNA expression normalized to *β-actin* (*n* = 5–6), and the right graph shows Cox7b protein expression normalized to β-actin (*n* = 9). All data are shown as means ± SEM. * *p* < 0.05, ** *p* < 0.01, *** *p* < 0.005, ns: *p* > 0.05; compared to MDA-MB-231 cells; using one-way ANOVA with Dunnett’s post hoc test (**a**–**d**).

*ALDH9A1* encodes a cytosolic aldehyde dehydrogenase that catalyzes the oxidation of γ-aminobutyraldehyde and aminoaldehydes derived from polyamines. It is involved in carnitine biosynthesis [44], which facilitates the transport of fatty acids across the inner mitochondrial membrane for β-oxidation and, potentially, in a marginal pathway for the biosynthesis of neurotransmitter γ-aminobutyric acid (GABA) [45] (Figure 3a, left). Compared to parental MDA-MB-231 cells, *ALDH9A1* mRNA expression was significantly increased in 231-BR, but it was slightly decreased in 231-BR-2 cells (Figure 3a, middle). The corresponding protein was overexpressed in 231-BR but not in 231-BR-2 cells (Figure 3a, right), which disqualified it as a shared metabolic sensor for brain tropism in our model cell lines.

*FH* encodes fumarate hydratase, the seventh enzyme of the TCA cycle that catalyzes the hydration of fumarate to *L*-malate (Figure 3b, left). When mutated/inactivated, FH can cause various diseases, including hereditary and sporadic forms of cancer [46]. In the context of brain-specific breast cancer metastasis, FH mRNA and protein expression was increased in 231-BR but not in 231-BR-2 cells (Figure 3b, middle and right), thus disqualifying this enzyme as a shared metabolic sensor for brain tropism.

*NDUFB8* encodes an accessory subunit of NADH ubiquinone oxidoreductase, a large protein complex known as ETC Complex I at the inner mitochondrial membrane (Figure 3c, left). The subunit is bound to NADH dehydrogenase 5 (ND5) in the proton-pumping module of Complex I [47]. Similar to microarray data analysis, RT-qPCR showed significantly reduced *NDUFB8* mRNA expression in 231-BR compared to MDA-MB-231 cells (Figure 3c, middle). However, it was significantly increased in 231-BR-2 cells, and the changes in protein expression did not match the changes in mRNA expression (Figure 3c, middle and right). Overall, this disqualified NDUFB8 as a metabolic sensor for brain-selective metastasis.

*COX7B* encodes subunit 7b of cytochrome c oxidase (Cox), a large protein complex known as ETC Complex IV that catalyzes the transfer of electrons from reduced cytochrome c to molecular oxygen at the inner mitochondrial membrane (Figure 3d, left) [48]. Cox7b is a short 80 amino acid protein that stabilizes the complex and modulates Cox activity [49]. *COX7B* mRNA expression was increased in 231-BR but decreased in 231-BR-2 cells (Figure 3d, middle). However, the expression of the corresponding protein was increased in both variants (Figure 3d, right). Considering that among the four candidate proteins only Cox7b expression showed a similar change in both brain-seeking variants, we retained Cox7b for further investigation. 

### 3.5. Cox7b Expression Drives Human Breast Cancer Cell Migration towards Astrocytes

Transwell migration assays were used to establish a causal link between Cox7b expression and MDA-MB-231 brain chemoattraction, mimicked by cell migration towards immortalized human and mouse astrocytes. Chemoattraction at other important metastatic sites [34] was mimicked by immortalized human hepatocytes (T0063) and human bronchial epithelial cells (T0763). All four cell lines were nonmalignant.

**Figure 4:**
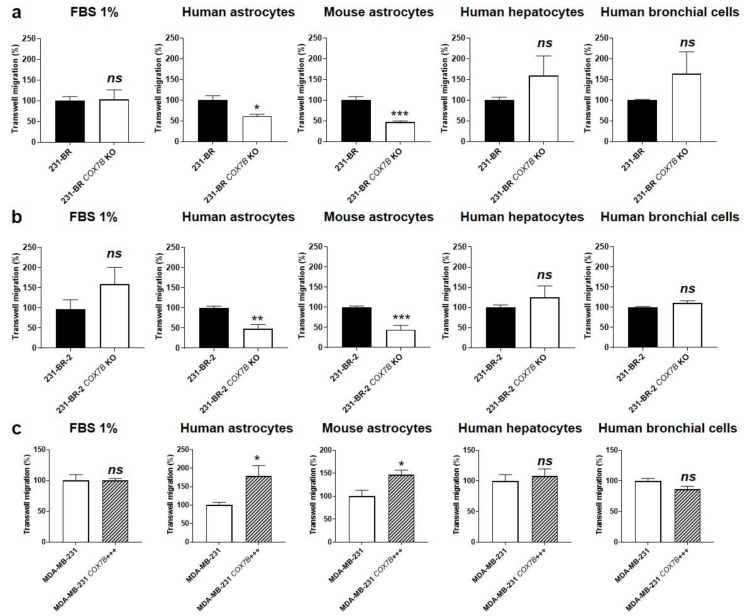
Cause–effect relationship between Cox7b expression and the selective migration of human breast cancer cells towards astrocytes. (**a**) Migration assayed in transwells of 231-BR brain-seeking variants, expressing or not expressing (KO using a CRISP-Cas9 strategy) Cox7b, towards 1% FBS (*n* = 9), human astrocytes (*n* = 3), mouse astrocytes (*n* = 5), human hepatocytes (*n* = 6), or human bronchial cells (*n* = 6). (**b**) Migration of 231-BR-2 brain-seeking variants, expressing or not expressing Cox7b, towards 1% FBS (*n* = 9), human astrocytes (*n* = 5–6), mouse astrocytes (*n* = 7), human hepatocytes (*n* = 6), or human bronchial cells (*n* = 6). (**c**) Migration of MDA-MB-231 parental cancer cells, overexpressing Cox7b or expressing basal levels of Cox7b, towards 1% FBS (*n* = 7), human astrocytes (*n* = 9), mouse astrocytes (*n* = 6), human hepatocytes (*n* = 6), or human bronchial cells (*n* = 6). All data are normalized to control (first columns) and are shown as means ± SEM. * *p* < 0.05, ** *p* < 0.01, *** *p* < 0.005, ns: *p* > 0.05; using Student’s t-test (**a**–**c**).

As expected, *COX7b* silencing in brain-seeking variant cells, using a CRISPR-Cas9 strategy (Figure S2a), significantly decreased 231-BR cell migration towards human and mouse astrocytes but not towards human hepatocytes and human bronchial cells (Figure 4a). The general migratory phenotype towards serum (1% FBS, used as a control) was unaffected. Similarly, *COX7B* silencing significantly reduced 231-BR-2 migration towards human and mouse astrocytes but not towards human hepatocytes, human bronchial cells, or serum (Figure 4b). Conversely, experimental Cox7b protein overexpression in parental MDA-MB-231 cells (Figure S2b) increased their migration towards human and mouse astrocytes, while migration towards human hepatocytes, human bronchial cells, or serum was not changed (Figure 4c).

Together, these in vitro results supported a cause–effect relationship between Cox7b protein expression and the brain tropism of human metastatic breast cancer cells. In particular, Cox7b protein overexpression was sufficient to trigger a selective brain tropism of otherwise pan-metastatic, wild-type MDA-MB-231 cells. This finding does not exclude that other proteins could have a similar function in other cancer cell lines. 

### 3.6. Cox7b Expression Promotes the Oxidative Phenotype of Human Metastatic Breast Cancer Cells 

The data displayed in Figure 2 showed that increased OXPHOS is a major metabolic characteristic of 231-BR and 231-BR-2 brain-seeking variants compared to parental cells. Since Cox7b resides in the ETC [49], we reasoned that its expression might modulate the OCR of the cells, which was measured using Seahorse oximetry.

**Figure 5:**
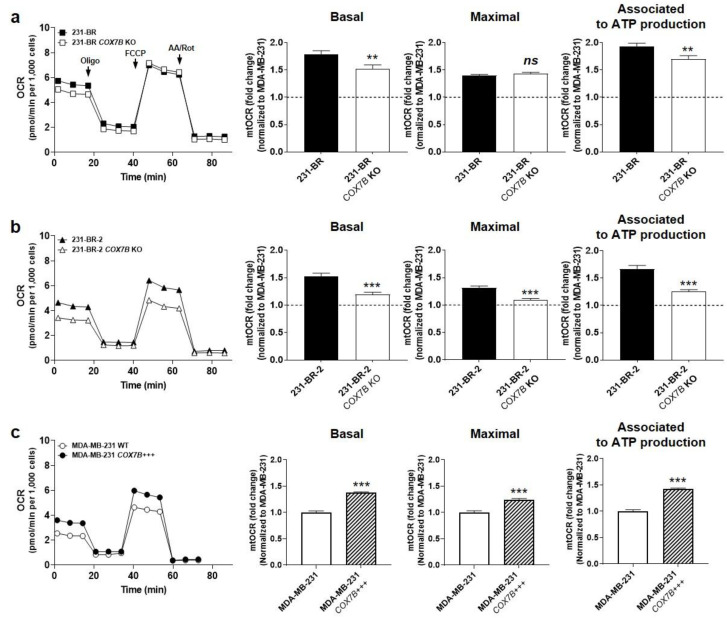
Cox7b expression drives the oxidative switch of brain-seeking variants. (**a**) 231-BR brain-seeking variants expressing or not expressing (KO using a CRISP-Cas9 strategy) Cox7b. Basal (**left**) and maximal (**middle**) oxygen consumption rates (mtOCRs), as well as the OCR of the cells associated to mitochondrial ATP production (**right**), were measured using a XF Cell Mito Stress Test Kit (Agilent) on a Seahorse XF96 bioenergetics analyzer (*n* = 20 all). Representative Seahorse traces are shown on far left. (**b**) As in (**a**), but using 231-BR-2 brain-seeking variants expressing or not expressing Cox7b (*n* = 15–20). (**c**) As in (**a**), but using MDA-MD-231 parental cancer cells overexpressing or expressing basal levels of Cox7b (*n* = 23–32). All data are normalized to control (dotted lines) and are shown as means ± SEM. ** *p* < 0.01, *** *p* < 0.005, ns: *p* > 0.05; using Student’s t-test (**a**–**c**).

*COX7B* silencing reduced 231-BR basal OCR and OCR associated to ATP production, but maximal OCR, reflecting the respiration spare capacity, was unchanged (Figure 5a). Comparatively, *COX7B* silencing reduced all basal OCR, maximal OCR, and OCR associated to ATP production in 231-BR-2 cells (Figure 5b), demonstrating that loss of *COX7B* represses OXPHOS in brain-seeking variants of metastatic breast cancer. Of note, *COX7B* silencing did not decrease cell numbers (Figure S3), suggesting the existence of rescue metabolic pathways preventing cell death. Cox7b overexpression in wild-type MDA-MB-231 cells induced the opposite effect, i.e., an oxidative switch characterized by a rise in all basal OCR, maximal OCR, and OCR associated to ATP production (Figure 5c).

Together, these experiments demonstrated that Cox7b is an OXPHOS inducer. They further established a positive correlation between the oxidative activities of human metastatic breast cancer cells and their preferential migration towards astrocytes. Of note, Cox7b expression did not modulate the OXPHOS substrate preference of the brain-seeking variants (Figure S4).

### 3.7. Cox7b Expression Is Responsible for the Brain Tropism of Metastatic Human Breast Cancer Cells in Mice

To experimentally establish a cause–effect relationship between Cox7b expression and breast cancer brain metastasis, we ran a series of in vivo experiments in nude mice, as depicted in Figure 1a. Briefly, because our investigation interrogated metastatic tropism linked to metastatic take (a late metastatic event) but not metastatic cell dissemination from a primary tumor (an early metastatic event), breast cancer cells were injected in the left cardiac ventricle, which is known to generate systemic metastatic lesions to the bones, brain, ovary, and adrenal glands using MDA-MB-231 cells [34,35]. The constitutive and concurrent expression of luciferase and GFP by our model cell lines allowed for a confirmation of bioluminescence data with immunohistochemistry.

**Figure 6:**
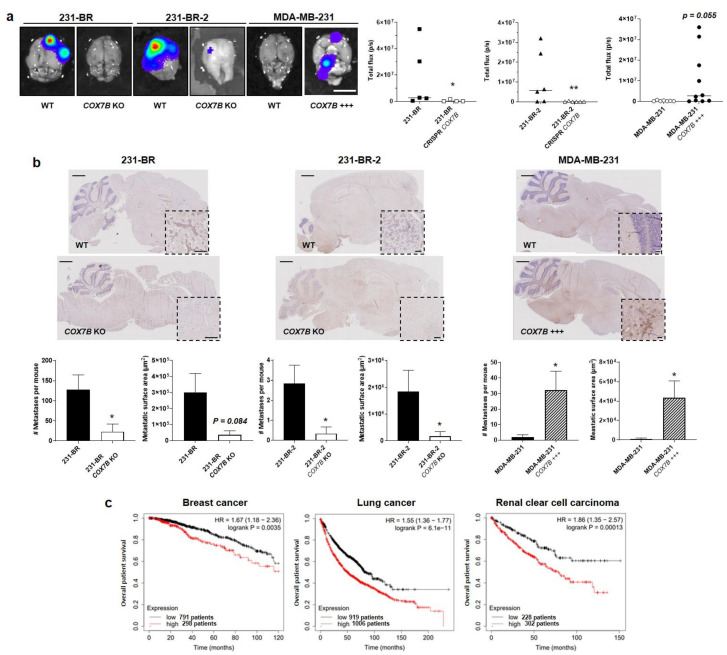
Cox7b drives the tropism of metastatic human breast cancer cells to the brain. (**a**,**b**) The brain tropism of 231-BR and 231-BR-2 brain-seeking variants, expressing or not Cox7b (KO using a CRISP-Cas9 strategy), and of parental MDA-MB-231 human breast cancer cells, overexpressing or not overexpressing Cox7b, was assessed using the protocol depicted in Figure 1a. The cells constitutively expressed luciferase and GFP. (**a**) Ex vivo bioluminescence imaging of mouse brains 4 weeks after the intracardiac injection of cancer cells. Pictures on the **left** are representative of mouse brains in luciferin-containing medium captured using a Xenogen IVIS 50 bioluminescence imaging system. Graphs on the right show brain bioluminescence intensity (*n* = 4–10). (**b**) Central sections of the brains were immunostained for GFP and counterstained for hematoxylin and eosin. Representative pictures are shown on top (bars = 1 mm). Insets represent typical GFP-positive metastatic lesions (bars = 100 µm). On the bottom, the left graphs represent the number of detected metastases (**left**) and the metastasis-positive surface area per mouse (*n* = 4–10). Representative images of the brains are shown. (**c**) Overall survival of patients with breast cancer (**left**, all types combined, 1090 patients), lung cancer (**middle**, all types combined, 1925 patients) and renal clear cell carcinoma (RCC, **right**, 530 patients). Data are shown as individual values and medians (**a**), means ± SEM (**b**), or individual values (**c**). * *p* < 0.05, ** *p* < 0.01, using Mann–Whitney test (**a**), Student’s t-test (**b**), or log rank test (**c**).

Following the protocol depicted in Figure 1a, *COX7B* silencing with a CRISPR-cas9 strategy in 231-BR and 231-BR-2 brain-seeking variants resulted in an almost total loss of brain tropism following intracardiac injection (Figure 6a). This was evidenced using ex vivo luciferase bioluminescence imaging on brains isolated at the time of mouse sacrifice. Conversely, parental MDA-MB-231 cells, which did not generate detectable brain metastasis for 4 weeks, gained a strong increase in the occurrence of brain metastases upon Cox7b overexpression (Figure 6a). For validation, brains were collected at the end of the experiments, sliced, stained with an antibody against GFP, and counterstained with hematoxylin and eosin. Figure 6b shows representative pictures of the brains, with insets representing typical metastasis-positive areas. Analyses revealed a strong decrease in the number of metastases per mouse and in the metastasis-positive tumor area per slice in mice injected with 231-BR and 231-BR-2 cells lacking *COX7B*, compared to wild-type 231-BR and 231-BR-2 cells (Figure 6b, left and middle graphs). The opposite effects were seen in mice that received parental MDA-MB-231 cells overexpressing Cox7b compared to wild-type MDA-MB-231 cells (Figure 6b, right graphs). Collectively, these in vivo data established a cause–effect relationship between Cox7b expression and the brain tropism of human TNBC in mice.

We concluded our study by analyzing publicly available gene chip and RNA-seq mRNA expression databases [43] reporting on clinical human breast and lung cancers, as wells as on renal clear cell carcinoma, all subtypes included. High Cox7B expression was identified as an independent poor prognosis factor for overall patient survival in all three cancer types (Figure 6c).

## 4. Discussion

In the context of the seed-and-soil hypothesis proposing that secondary organs should fulfill the specific needs of metastatic progenitor cells [14], this study aimed to investigate the existence of a metabolic control of tissue-specific metastasis. We used breast cancer brain metastasis as an example, and we selected the human MDA-MB-231 TNBC cancer cell line as a working model for the generally high propensity of this breast cancer subtype to metastasize in humans [50] and because two brain-seeking variants derived from the same parental cell line were already available from two independent laboratories [34,35,36]. It allowed us to validate characteristics identified in one variant by those of the other. In this model, we report that Cox7b, a structural subunit of ETC Complex IV [51,52], drives metastatic breast cancer cell homing to the brain: repression of Cox7b expression selectively blocked the migration of brain-seeking variants towards astrocytes and their capacity to generate brain metastases in mice; conversely, pan-metastatic parental cells manipulated to gain Cox7b expression increased their selective migration towards astrocytes and their capability to generate brain metastases. This series of experiments established a cause–consequence relationship between Cox7b expression and metastatic brain tropism in the MDA-MB-231 model. Other proteins could exert a similar function in other cancer cell lines and models.

Mammalian Cox, also known as Complex IV, is a 13-subunit multiheteromeric enzyme that catalyzes the oxidation of cytochrome c and the reduction of molecular oxygen to water at the terminal step of OXPHOS in the mitochondrial ETC [48]. Complex IV is located at the inner mitochondrial membrane. In the complex, Cox7b is a short 80 amino acid nuclear-encoded transmembrane protein that associates with mitochondria-encoded subunits Cox1, Cox2, and Cox3, which contain the four catalytic redox centers of the enzyme [49]. Cox7b is ubiquitously expressed. It has no enzymatic activity but stabilizes the complex and positively modulates Cox activity. Its expression is increased in several degenerative pathologies characterized by high OXPHOS activities (see reference [53] for a recent review), and inactivating mutations have been associated with the development of microphthalmia with linear skin lesions (MLS) [54]. The fact that Cox7b stabilizes Complex IV is in line with our observation that high Cox7b expression triggers OXPHOS, whereas *COX7B* silencing has the opposite effect. To date, nothing is known about the regulation of Cox7b expression in mammalian cells.

Interestingly, high Cox7b expression was enhanced through rounds of in vivo selection using intracardiac delivery in mice, thus bypassing adaptation and selection in a primary tumor. This highlights a key characteristic of metastatic cancer cells: selective homing. Homing is an active process involving cancer cell interactions with vascular endothelial cells at given body locations; transvascular diapedesis; nesting in the premetastatic niche; the establishment of molecular relationships with host cells at the new location; and a phenotypic reversion from a stem to a proliferative phenotype for most post-metastatic cancer cells [26,55]. This succession of events would be incomplete without answering to the question: how do metastatic progenitor cells sense that they have arrived at the metastatic location, while they are still in the blood stream? If they exist, sensor systems should be sensitive enough to discriminate changes in the composition of the blood between different organs.

For homing, physical interactions between metastatic progenitor cells and host cells in the metastatic niche can be excluded as a triggering event, because they occur after extravasation. Theses interactions would rather primarily retain and re-educate/redifferentiate cancer cells at the metastatic site. Similarly, physical interactions between metastatic progenitor cells and endothelial cells lining blood vessels along premetastatic niches might not be the primary event for homing. Indeed, our in vitro experiments demonstrate that selective cancer cell migration towards host cells, seen as feeder cells, can be manipulated in the absence of vascular cells in vitro. In other words, metastatic progenitor cells would sense soluble molecules produced by host cells that act as chemoattractants. These molecules are expected to reach the blood stream in concentrations high enough to be sensed and with a concentration gradient steep enough to be followed by metastatic progenitors. Our data suggest that Cox7b could be such a sensor in human TNBC cells, but the exact nature of the metabolic signal(s) recognized by Cox7b is still unknown. Based on the observation that Cox7b expression increases OXPHOS activity, candidate chemoattractants should be primarily sought among TCA cycle substrates and intermediates. These chemoattractants would be produced by human and mouse astrocytes in culture and in vivo, but not by human bronchial cells nor by human hepatocytes. Lactate, which is produced and secreted by astrocytes to feed neurons [56], pyruvate, acetate, and glutamate are attractive candidates [57,58]. Of note, brain-seeking variants also gained metabolic flexibility to fuel OXPHOS, but this was not linked to Cox7b expression.

Our study was primarily aimed at proving the concept of a metabolic control of organotropism, and our data support that idea, even if additional experiments are still warranted to demonstrate our initial hypothesis. Cox7b was identified using the MDA-MB-231 model solely. This TNBC cell line was used because it represents a cancer type that is often detected in patients before entry in the metastatic phase, but that evolves to this phase despite treatments in a significant number of patients. Hence, in a cohort study where 25,362 TNBC patients were included, only 6% were at the metastatic stage and only 0.68% had brain metastases at the time of diagnosis; however, even for those with localized disease, approximately 25% of patients relapsed with distant metastasis [59,60]. Overall, brain metastasis affected up to 50% of TNBC patients in the course of the disease [10]. Our choice of the model was, thus, driven by the possibility to identify a suitable target for the prevention of brain metastasis. At the end of the study, one must recognize that Cox7b is not a suitable pharmacological target, as this structural protein has no enzymatic activity and is buried within Complex IV at the mitochondrial inner membrane. However, we believe that the use of the same selection approach for in vivo organotropism starting from different types of cancer cells (e.g., other breast cancer cells, prostate cancer, cervix cancer, melanoma) will reveal additional metabolic proteins controlling tissue-specific homing. Candidates have already been proposed [61] that must still be validated as being causal in organotropism. Among these, some could be amenable for therapy, with the ultimate intention of interfering with the metabolic sensing of different subtypes of metastatic progenitor cells at different secondary sites. Some of these targets could further be shared by different types of tumors (as illustrated here, with high Cox7b expression in primary tumors being a poor predictive factor of overall survival not only in breast cancer but also in lung cancer and in renal clear cell carcinoma), and others could be specific for a particular cancer type metastasizing to a particular secondary organ. Furthermore, a metabolic sensor could have additional effects unrelated to metastasis. This is the case of Cox7b, which sensitizes cancers to cisplatin chemotherapy, with high Cox7b expression in primary tumors being a favorable factor for overall patient survival upon cisplatin treatment [62].

## 5. Conclusions

This study is the first of a series investigating the existence of metabolic sensors for tissue-specific metastasis in the context of the seed-and-soil hypothesis and the premetastatic niche theory. Using parental human MDA-MB-231 TNBC cells and two independent brain-seeking variants selected in mice as models, we identified mitochondrial protein Cox7b in ETC Complex IV as a selective regulator of brain metastasis. Silencing and overexpression experiments established a causal link between Cox7b expression and metastatic brain tropism in vivo, where metabolically active astrocytes were sufficient to chemoattract brain-seeking metastatic variants expressing high levels of Cox7b. While Cox7b is not adapted as a direct target for the therapeutic prevention of brain metastasis, we believe that our general strategy, applied to other cancer types and/or different secondary sites, has the potential to unravel other, unprecedented target candidates.

## 6. Patent

M.C.N.M.B. and P.S. are inventors of patent application EP22191920, entitled “Stem Cells with Brain Tropism”, which is related to the work reported in this manuscript.

## Figures and Tables

**Table 1:** Metabolic genes differentially expressed in 231-BR versus other tissue-specific variants of parental MDA-MB-231 human breast cancer cells.

Gene ID	231-BR (Brain-Seeking)	ADMD-231 (Adrenal-Glands-Seeking)	BMD-231 (Bone-Seeking)	LMD-231 (Lung-Seeking)	*p* ^4^	*p* ^5^
*HK2* ^1^	**1.62** ^2,3^	**1.83**	**1.66**	**1.70**	>0.05	>0.05
*ALDH1A3*	1.18	**1.53**	**1.43**	**1.48**	<0.05	>0.05
*ALDH9A1*	**1.55**	1.04	1.07	1.09	>0.05	<0.05
*ALDH3A1*	**1.18**	1.03	1.21	1.16	>0.05	>0.05
*PCK2*	1.08	−1.43	**−1.21**	**−1.33**	<0.05	<0.05
*IDH1*	−1.18	**−1.31**	−1.13	−1.14	>0.05	>0.05
*FH*	**1.54**	1.08	1.04	1.11	>0.05	<0.05
*MDH2*	−1.10	**−1.19**	**−1.17**	−1.27	>0.05	>0.05
*ATP5I*	1.25	**1.36**	**1.33**	1.33	>0.05	>0.05
*ATP5G2*	−1.06	**−1.21**	−1.07	−1.11	>0.05	>0.05
*ATP6V0E2*	**1.62**	1.43	1.41	1.34	>0.05	>0.05
*ATP6V1D*	1.69	**2.45**	1.83	1.96	>0.05	>0.05
*ATP6V0D1*	−1.02	**−1.23**	−1.14	−1.11	>0.05	>0.05
*ATP6V0D2*	**1.21**	1.08	1.03	1.20	>0.05	>0.05
*ATP6V1B1*	**1.25**	−1.09	−1.07	1.03	<0.05	<0.05
*COX17*	−1.47	−1.46	**−1.50**	−1.52	>0.05	>0.05
*COX7B*	**2.02**	1.06	−1.06	−1.20	<0.05	<0.05
*NDUFB7*	1.45	**1.54**	1.27	1.21	>0.05	>0.05
*NDUFB8*	**−1.43**	**1.46**	**1.45**	**1.54**	<0.05	<0.05
*NDUFV3*	1.10	**1.51**	1.42	1.37	>0.05	>0.05
*UQCRB*	**1.31**	1.05	1.27	1.30	>0.05	>0.05
*PGM5*	−1.11	**6.42**	**4.32**	**4.51**	<0.05	<0.05

^1^ From the GEO #GSE66495 microarray database (*n* = 3 per cell line), including only those genes involved in glycolysis, the TCA cycle, and oxidative phosphorylation. ^2^ Numbers report on fold changes, compared to parental MDA-MB-231 cells used as control. ^3^ Bold numbers are fold changes with *p* < 0.05 compared to parental MDA-MB-231 cells using one-way ANOVA. ^4^ *p* value for 231-BR compared to all other tissue-seeking variants using one-way ANOVA. ^5^ *p* value for 231-BR compared to all other tissue-seeking variants and parental cells using one-way ANOVA. Genes of interest (*p* < 0.05) are highlighted in gray.

## Data Availability

All data are contained within the article and the Supplementary Materials.

## Supplementary Materials

The following supporting information can be downloaded at: https://www.mdpi.com/article/10.3390/cancers14184371/s1. Figure S1: uncropped western blots; Figure S2: Validation of *COX7B* silencing and overexpression; Figure S3: *COX7B* silencing does not alter brain-seeking variant cell numbers in vitro; Figure S4: *COX7B* silencing does not alter the metabolic plasticity of brain-seeking variant cells; Table S1: Short tandem repeat profiles.

## Appendix A

### Appendix A.1. Cell Infection for Constitutive Luciferase and GFP Expression

For constitutive luciferase and GFP expression, cells were infected with lentiviruses carrying the luciferase and GFP sequences along with a puromycin resistance gene (Amsbio; catalogue #LPV020). Briefly, 70–80% of the confluent cells in a 24-well plate were transduced with 2 μL per well of the lentivirus solution in 1mL medium with 10 μL/mL polybrene. Cells were selected by a 48–72 h incubation with 1 µg/mL puromycin (InvivoGen, Toulouse, France) and FACS-sorted for GFP expression on a Becton Dickinson FACSAriaIII system (Erembodegem, Belgium).

### Appendix A.2. COX7B Gene Silencing

CRISPR plasmids were constructed following Zhang’s laboratory protocol [37] with pX459 (Addgene; catalogue #62988, puromycin selection) or pU6-(BbsI)_CBh-Cas9-T2A-mCherry (Addgene; catalogue #64324, red fluorescence selection) plasmids [37,63,64]. These plasmids contain both Cas9 and guide RNA (gRNA) expression cassettes, with BbsI restriction sites for the insertion of gRNA sequences. Prevalidated gRNA sequences were chosen in the GenScript genome-wide database [65] for a non-overlapping duo of gRNAs: 5′-AGCGCACTAAATCGTCTCCA-3′ and 5′-GAGTTACCCCAAAGGAATGG-3′. Sticky ends were created for insertion in the vector plasmids, with CACCG at the 5′ of the gRNA sense sequence and AAAC at the 5′ and C at the 3′ of the gRNA antisense sequence.

gRNA oligonucleotides (Eurogentec, Seraing, Belgium) were annealed into double-stranded DNA, with 1 µL of stock solutions containing 100 µM of each sense and antisense oligonucleotides in 2 µL of 5X T4 ligase buffer (Thermo Fisher; catalogue #46300018), 0.5 µL of T4 PNK (BIOKE; catalogue #M0201) and 5.5 µL of DNase/RNase-free distilled water. Incubation times were 37 °C for 30 min, followed by 95°C for 5 min, and then the temperature was decreased at a rate of 5 °C/min until reaching 25 °C.

The Golden Gate DNA Assembly protocol [66] was then used for inserting 1 µL of annealed gRNA at 1 µM into 100 ng of vector plasmid, in a solution containing 5 µL of 10x Fast Digest buffer (Thermo Fisher; catalogue #B64), 0.5 µL of ATP 0.1 M (Thermo Fisher; catalogue #R1441), 0.5 µL of BSA 10 mg/mL (Promega; catalogue #R396D), 1 µL of restriction enzyme BpiI (Thermo Fisher; catalogue #FD1014), and 2 µL of T4 ligase 5 U/µL (Thermo Fisher; catalogue #EL0014) in a total volume of 50 µL completed with water. The mixture was incubated for 20 cycles at 37 °C for 5 min and 20 °C for 5 min, followed by 80 °C for 20 min.

Five microliters of the resulting solution were used for TOP10 bacteria (Thermo Fisher; catalogue #C404003) transformation using prewarmed LB agar plates (Thermo Fisher; catalogue #22700-025) containing 100 µg/mL of ampicillin, in accordance with the instructions of the manufacturer. Single colonies were inoculated in LB broth (Thermo Fisher; catalogue #12780-052) with 50 µg/mL ampicillin and incubated at 37 °C overnight. Plasmid DNA was then collected with the PureYield Plasmid Miniprep System (Promega; catalogue #A1223), and its concentration was obtained using a NanoDrop device (Thermo Fisher). Sequences were verified by Sanger Sequencing (Genewiz, Leipzig, Germany).

Cancer cells at 70–80% confluence were transfected with the Lipofectamine LTX/Plus transfection kit (Thermo Fisher; catalogue #15338100) or with the jetOPTIMUS kit (Westburg, Leusden, The Netherlands; catalogue #117-01). The first kit was used with 0.25 µg of each gRNA plasmid, for a total of 0.5 µg of DNA in 100 µL of OptiMEM, containing 0.5 µL of Plus and 2.25 µL of lipofectamine LTX, with incubation times of 15 and 30 min, respectively. The second kit was used with 0.5 µg of each gRNA plasmid, for a total of 1 µg of DNA, with 1 µL of reagent in 200 µL of buffer for each well in a 6-well plate. Cells were selected with puromycin (1 μg/mL)-containing medium for 48–72 h or FACS-sorted for mCherry fluorescence 48 h after transfection on a Becton Dickinson FACSAriaIII system.

### Appendix A.3. Cox7b Overexpression

The human untagged *COX7B* cDNA ORF Clone in expression vector pCMV3 (Bio-Connect; catalogue #HG20762-UT) was used to overexpress Cox7b in MDA-MB-231 cancer cells. Cells at 70–80% confluence were transfected with 10 µg of the plasmid and Lipofectamine 3000 (Thermo Fisher; catalogue #L3000001) in a 10 cm dish, allowed to recover the next day in fresh DMEM containing glutaMAX and 4.5 g/L glucose (Thermo Fisher; catalogue #61965026) supplemented with 10% FBS, and selected with hygromycin (400 µg/mL)-containing medium for 10 days. Medium was renewed every 3–4 days. Colonies were individually picked, expanded, and tested by western blotting for the expression of Cox7b.

### Appendix A.4. Cell Migration and Invasion

Invasion was assessed by coating the inserts with 250 μg/mL of Matrigel (Corning, Tewksbury, MA, USA; #356231) for 2 h at 37 °C, and migration without Matrigel coating. Fifty thousand cancer cells were seeded in the upper chamber of each transwell in 500 µL of serum-free culture medium, while the lower chamber contained FBS 1% (general migration/invasion) or confluent nonmalignant astrocytes, hepatocytes, or bronchial cells, which were used as attractants. Cancer cells were allowed to migrate/invade for 24 h at 37 °C in a 5% CO_2_ humidified atmosphere. At the end of the assay, cells were fixed with 4% PFA in PBS for 10 min, rinsed three times with PBS, and immobile cells (upper compartment of the insert) were wiped away. Mobile cells were stained with DAPI for 30 min, rinsed three times with PBS, and imaged at 5x on an AxioVert microscope equipped with an AxioCam-MRc camera (Zeiss). Two images were taken per well, thus covering the upper and lower halves. Nuclei were counted using QuPath software version 0.1.2. with the Positive Cell Detection analysis tool.

### Appendix A.5. Seahorse Oximetry

Basal, maximal, and ATP-linked oxygen consumption rates (OCRs) were quantified using the XF Cell Mito Stress Test Kit (Agilent) on a Seahorse XF96 bioenergetics analyzer (Agilent). Briefly, 10,000 cells were seeded in XF96 culture plates 16 h before the experiments in DMEM, containing glutaMAX and 4.5 g/L glucose (Thermo Fisher; catalogue #61965026) supplemented with 10% FBS. On the day of analysis, the culture medium was replaced by DMEM containing 10 mM glucose, 2 mM glutamine, 1.85 g/L NaCl, and 3 mg/L phenol red, pH 7.4. Cells were further incubated for 1 h in a CO_2_-free incubator before analysis. Sequentially, basal OCR was acquired without treatment, ATP-linked OCR after the addition of 1 µM of ATP synthase inhibitor oligomycin, maximal OCR after mitochondrial potential disruption using 1 µM of ionophore carbonyl cyanide-4-(trifluoromethoxy)phenylhydrazone (FCCP), and non-mitochondrial OCR after the addition of 0.5 µM of Complex I inhibitor rotenone together with 0.5 µM of Complex III inhibitor antimycin A.

Mitochondrial fuel dependency was determined using the Fuel Flex test kit (Agilent) with either 2 µM of mitochondrial pyruvate carrier (MPC) inhibitor UK5099, 3 µM of glutaminase inhibitor BPTES, or 4 µM of carnitine palmitoyltransferase-1 (CPT-1) inhibitor etomoxir, to challenge oxidative glucose metabolism, glutaminolysis, and lipolysis, respectively.

Mitochondrial OCRs (mtOCRs) were calculated by subtracting non-mitochondrial OCRs from the corresponding basal, maximal, and ATP-linked OCRs. Respiration used for ATP production is the difference between basal OCR and OCR in the presence of ATP-synthase inhibitor oligomycin. All data were normalized by the total protein content.
