# Supplementary material for: Mitochondrial Protein Cox7b Is a Metabolic Sensor Driving Brain-Specific Metastasis of Human Breast Cancer Cells
Source: Cancers (Basel). 2022 Sep 8;14(18):4371. doi: 10.3390/cancers14184371 (PMC9497206; doi:10.3390/cancers14184371)
Supplement: Supplementary file 1 [file cancers-14-04371-s001.zip › cancers-1881530-supplementary.pdf]

Table S1. Short tandem repeat profiles.

STRs	MDA-MB-231	231-BR	231-BR-2
D8S1179	13, 13	13, 13	13, 13
D21S11	30, 33.2	30, 33.2	30, 33.2
D7S820	8, 9	8, 8	8, 8
CSF1PO	12, 13	12, 13	12, 13
D3S1358	16, 16	16, 16	16, 16
TH01	7, 9.3	7, 9.3	7, 9.3
D13S317	13, 13	13, 13	13, 13
D16S539	12, 12	12, 12	12, 12
D2S1338	20, 21	20, 21	20, 21
D19S433	11, 14	11, 14	11, 14
vWA	15, 18	15, 16	15, 18
TPOX	8, 9	8, 9	8, 9
D18S51	11, 16	11, 15, 16	16, 16
AMEL	X, X	X, X	X, X
D5S818	12, 12	12, 12	12, 12
FGA	22, 23	22, 23	22, 23

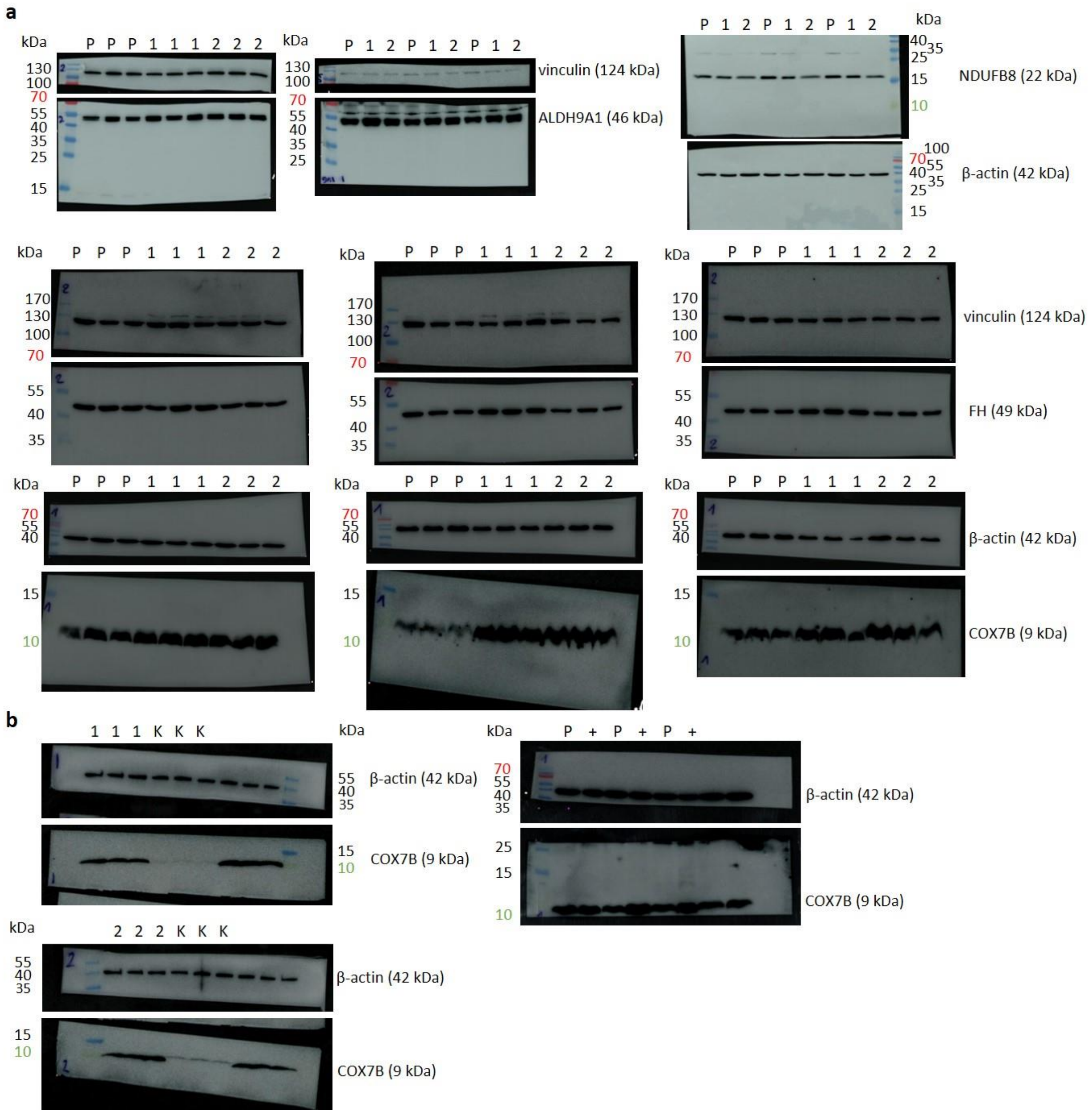

Figure S1. Uncropped western blot images. (**a**, **b**) Shown are whole western blot membranes corresponding to (**a**) Figure 3a-d, and (**b**) Figure S2. Note that, in order to be able to show molecular ladders, the membranes are shown at the longest exposure time (often overexposed), which does not always correspond to the acquisition time on an ECL imager 600. Abbreviations: P, parental; 1, 231-BR; 2, 231-BR-2; K, KO cells for *COX7B*; +, cells overexpressing COX7b.

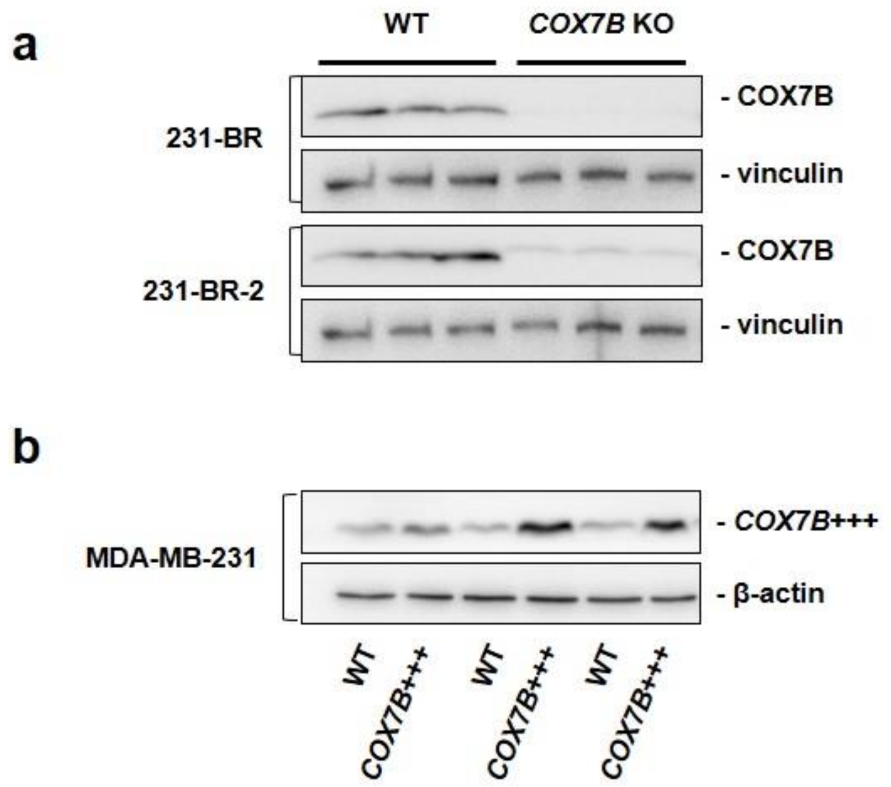

Figure S2. Validation of *COX7B* silencing and overexpression. (**a**) *COX7B* was silenced in 231-BR and 231-BR-2 brain-seeking variants using a CRISPR-Cas9 strategy. Shown are western blots reporting in triplicate on COX7b expression. Vinculin served as a loading control. (**b**) COX7b was overexpressed in parental MDA-MB-231 cells using a pCMV3 vector. Shown are western blots reporting in triplicate on COX7b expression. β-actin served as a loading control.

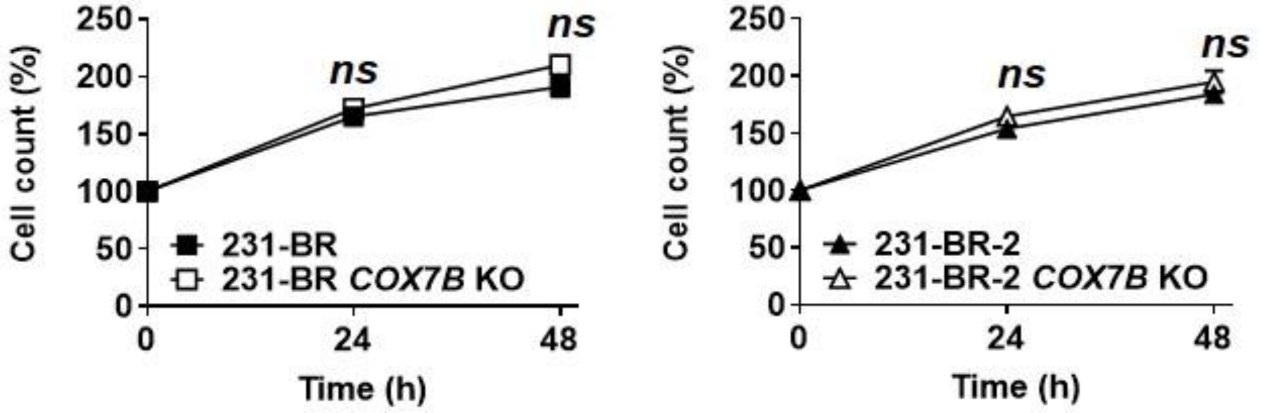

Figure S3. *COX7B* silencing does not alter brain-seeking variant cell numbers *in vitro*. 231-BR and 231-BR-2 cells were either wild-type or silenced for *COX7B* using a CRISPR-Cas9 strategy. Graphs show cell count (%) over time on a SpectraMax i3 spectrophotometer equipped with a MiniMax imaging cytometer, after seeding 5,000 cells per well in a 96-wells plate (n = 3-4). Data are shown as means ± SEM. *ns*: $p > 0.05$ compared to MDA-MB-231 cells; using two-way ANOVA with Dunnett's post hoc test.

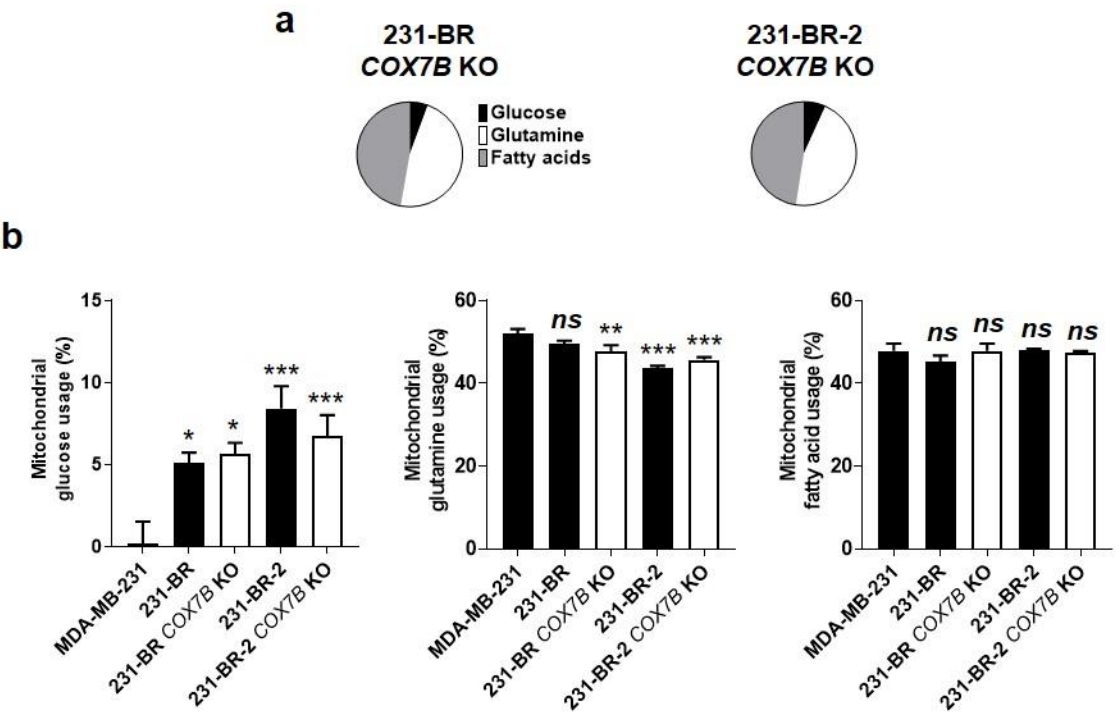

Figure S4. *COX7B* silencing does not alter the metabolic plasticity of brain-seeking variant cells. (**a, b**) 231-BR and 231-BR-2 cells were either wild-type or silenced for *COX7B* using a CRISPR-Cas9 strategy. (**a**) Mitochondrial fuel usage of *COX7B*-deficient 231-BR (left, n = 7-14) and *COX7B*-deficient 231-BR-2 (right, n = 12-16) cancer cells was determined using the Fuel Flex test kit (Agilent) on a Seahorse XF96 bioenergetics analyzer. Data are presented as pie graphs. (**b**) Mitochondrial fuel usage of parental MDA-MB-231 cells, 231-BR cells expressing or not expressing Cox7b and 231-BR-2 cells expressing or not expressing COX7b. Column graphs show mitochondrial glucose (left; n = 7-15), glutamine (middle; n = 12-16) and fatty acid (right; n = 14-16) usage, where total fuel (glucose + glutamine + fatty acids) usage = 100% for each cell line. Data are shown as means ± SEM. * $p < 0.05$, ** $p < 0.01$, *** $p < 0.005$, *ns*: $p > 0.05$ compared to MDA-MB-231 cells; using one-way ANOVA with Dunnett's post hoc test.
